# Assessment of Exocrine Pancreatic Function Following Bariatric/Metabolic Surgery: a Prospective Cohort Study

**DOI:** 10.1007/s11695-022-06359-4

**Published:** 2022-11-28

**Authors:** Gülten Çiçek Okuyan, Doğukan Akkuş

**Affiliations:** Department of General Surgery, Haydarpaşa Numune Education and Training Hospital, Tıbbiye Cad. No: 23, 34668 Istanbul, Turkey

**Keywords:** Exocrine pancreatic insufficiency, Pancreatic elastase, Quality of life, Bariatric surgery

## Abstract

**Background:**

Exocrine pancreatic insufficiency (EPI) can be seen after bariatric/metabolic surgery. Fecal elastase level is a simple test in diagnosing and grading EPI. Quality of life changes in patients with bariatric/metabolic surgery related to gastrointestinal complaints is debated.

**Aim:**

This study aimed to investigate rates and grades of EPI via fecal elastase levels and association between EPI and quality of life in bariatric surgery patients.

**Methods:**

A prospective study was performed for patients with bariatric/metabolic surgery at their second-year follow-up. Fecal elastase levels were used to diagnose and grade EPI as severe or moderate. Patient’s gastrointestinal quality of life index (GIQLI) was calculated. Patients were grouped as sleeve gastrectomy (SG), one-anastomosis gastric bypass (OAGB), single-anastomosis sleeve ileal bypass (SASI), and transit bipartition (TB). Rates of severe or moderate EPI were primary outcome. Secondary outcome was an association between fecal elastase and GIQLI.

**Results:**

There were 17, 29, 21, and 15 patients in OAGB, SG, TB, and SASI groups. There was no significant difference between groups in GIQLI scores and fecal elastase levels (*p* = 0.152 and *p* = 0.361). Rates of patients with moderate EPI in the groups OAGB, SG, TB, and SASI were 23.5%, 17.2%, 14.3%, and 20.0%. GIQLI scores were not significantly correlated with age, postoperative morphometric data, and fecal elastase values (*p* > 0.05).

**Conclusion:**

Rates of patients with moderate EPI ranged from 14.3 to 23.5% at second-year follow-up. There was no patient with severe EPI. GIQLI scores were not significantly correlated with fecal elastase levels and different types of bariatric/metabolic surgery.

**Graphical Abstract:**

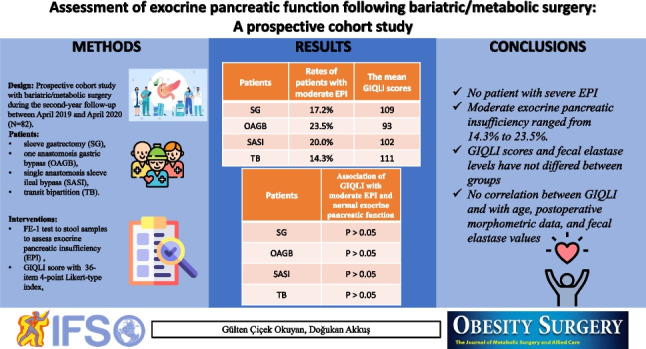

## Introduction

Any disturbance of the exocrine pancreatic function leading to maldigestion is defined as exocrine pancreatic insufficiency (EPI) [1]. Although various pancreatic and extrapancreatic diseases have been speculated to cause EPI, pancreatic and gastrointestinal operations are reportedly the major predisposing factors [1, 2]. The possible impact of bariatric surgery on EPI has also been addressed in the literature [2–4]. The amount of biliopancreatic secretions may vary depending on the type of bariatric surgery [1, 5]. Vitamin and mineral deficiencies can also be experienced after bariatric surgery depending on the length of the bypassed proximal intestine [2, 4, 5]. That being said, asynchrony between gastric emptying and biliopancreatic secretion after bariatric surgery has been reported as the main triggering factor for EPI in this patient group [1, 6]. However, previous studies reported controversial rates of EPI following different bariatric surgical modalities [1, 5].

Direct measurement of molecules such as cholecystokinin and secretin secreted by the exocrine pancreas has been used to diagnose EPI [7]. The difficulty in obtaining the pancreatic fluid to be used in these measurements is the main factor that limits the widespread use of the direct measurement of molecules secreted by the exocrine pancreas in diagnosing EPI [1, 7]. Therefore, fecal fat quantification remains the gold standard for diagnosing EPI [2, 4]. However, the technical difficulties of fecal fat quantification in respect of both the patients and the laboratory staff drive the research on simpler and more indirect methods that can be used to measure pancreatic exocrine function. In this context, quantification of fecal elastase (fecal elastase-1) has been offered as a non-invasive method to diagnose PEI in several patient groups [1, 2, 4, 5, 7–9]. Although the inverse relationship between fecal elastase levels and the risk of EPI has been known, several studies reported controversial findings about the predictive power of fecal elastase for EPI [2, 10].

Bariatric surgery has been regarded as the most efficient treatment modality for obesity [1, 5]. Although bariatric surgery reportedly leads to significant improvements in gastrointestinal complaints due to severe obesity, some of these patients can develop gastrointestinal symptoms associated with fat maldigestion, fat malabsorption, and EPI in the postoperative period [6, 11]. Hence, there is an ongoing debate about the potential impact of gastrointestinal disturbances on the quality of life of these patients [12]. Several questionnaires, e.g., the Bariatric Analysis and Reporting Outcome System (BAROS) and the gastrointestinal quality of life index (GIQLI), have been proposed to be used on patients who underwent bariatric surgery to evaluate the quality of life of these patients in the postoperative period [4, 12]. The use of GIQLI, one of these questionnaires, after bariatric surgery has not been studied in detail [12, 13].

In this context, this study aimed to evaluate the rates of EPI in patients who underwent different types of bariatric/metabolic surgery during their second-year follow-up and investigate the relationship between EPI and the quality of life using the GIQLI.

## Materials and Methods

### Research Design

This study was designed as a prospective cohort study. The study protocol was approved by the local ethical committee (Haydarpasa Education and Research Hospital Ethical Committee for Clinical Studies, HNEAH-KEAK 2021/KK/139). The study was performed in accordance with the principles set forth in the Declaration of Helsinki. The written informed consent was taken from the patients.

### Population and Sample

The population of the study consisted of patients who underwent bariatric/metabolic surgery in a tertiary hospital, i.e., Haydarpasa Education and Research Hospital, Department of General Surgery, in Istanbul, Turkey, between April 2019 and April 2020 due to obesity and metabolic diseases and were at their second-year follow-up.

The criteria recommended by the International Federation for the Surgery of Obesity and Metabolic Disorders (IFSO) that is either having a body mass index (BMI) value of > 40 kg/m^2^ or having a BMI value of > 30–35 kg/m^2^ and an inadequately monitored metabolic disease/being treated for a metabolic disease were used to diagnose obesity [14]. Patients with a history of pancreatitis or previous pancreatic or upper gastrointestinal surgery, concomitant use of pancreatic enzymes, laxatives, motility agents, and major vascular events within the last six months were excluded from the study. The types of bariatric surgery that the patients underwent were sleeve gastrectomy (SG), one anastomosis gastric bypass (OAGB), single anastomosis sleeve ileal bypass (SASI), and transit bipartition (TB). The respective technical aspects of the said surgical procedures have been described previously in the literature [12, 15–18]. All surgeries were performed by a single bariatric surgeon with at least 10 years of experience. The type of surgery patients undergo was left to the surgeon’s discretion. Nevertheless, the general indications that the surgeon considered while deciding on the type of the surgery were as follows: SG was preferred in young and non-diabetic patients, OAGB or SASI was preferred in patients with class III obesity and non-diabetic patients, and OAGB, SASI, or TB was preferred in patients with diabetes and C-peptide levels over 1.5 ng/ml.

### Interventions

#### FE-1 Test

The stool samples to be used in the FE-1 test were collected with the sample preparation kit (Biometric Services (BIOSERV)) containing the extraction and stored at − 20 °C. The patients were requested to stop taking proton pump inhibitors or non-steroidal anti-inflammatory drugs at least 3 days before feces collection [9].

The stool samples were brought to room temperature on the day of analysis, defrosted, centrifuged, and further sampled from the supernatant portions. Fecal elastase levels were evaluated with the enzyme-linked immunosorbent assay kit (BIOSERVE Diagnostics GmbH, Rostock, Germany). The results were reported in micrograms of elastase per gram of stool (µg/g). Results < 100 µg/g, 100 to 200 µg/g, and > 200 µg/g were deemed to indicate severe EPI, moderate EPI, and normal exocrine pancreatic function, respectively [5, 6, 9].

### GIQLI

GIQLI was used to evaluate patients’ quality of life regarding their gastrointestinal symptoms [19]. GIQLI is a 36-item 4-point Likert-type index. The validity and reliability studies of the Turkish version of GIQLI have been performed [20]. GIQLI score was calculated for each patient included in the study. GIQLI evaluates the patient’s last 2 weeks regarding physical function (seven items), emotional function (five items), social function (five items), and gastrointestinal symptoms (19 items). Each item is assigned a score from zero to four points. The total GIQLI score is calculated by adding the scores assigned to each item. Accordingly, the total GIQLI score can be between zero and 144. The higher the total GIQLI score, the better quality of life.

The specific gastrointestinal complaints associated with EPI, including abdominal pain, flatulence, diarrhea, constipation, and reflux, were also investigated within the scope of the study [5, 11].

### Variables

Patients’ demographic (age, gender) and clinical data were obtained from the hospital records. Patients’ preoperative and postoperative anthropometric parameters, including weight (kg), height (m), and BMI (kg/m^2^) values, were recorded. The BMI change (%) and percent excess weight loss (%EWL) were calculated using these data. The information on the type of surgery patients underwent was obtained from the medical records and recorded. The laboratory analysis included measuring fecal elastase levels (µg/g) 2 years after the surgery. The gastrointestinal complaints of the patients (abdominal pain, flatulence, diarrhea, constipation, and reflux) were recorded [21].

### Study Groups

The patients were grouped according to the type of bariatric surgery they had as groups SG, OABG, SASI, and TB.

### Statistical Analysis

The primary and secondary outcomes of the study were the rates of patients with severe or moderate EPI and the relationship between fecal elastase levels and GIQLI scores, respectively.

Descriptive statistics were expressed as mean ± standard deviation values in the case of continuous variables that were determined to conform to the normal distribution, as median with minimum–maximum values in the case of continuous variables that were determined not to conform to the normal distribution, and as numbers and percentage values in the case of categorical variables. The Shapiro–Wilk, Kolmogorov–Smirnov, and Anderson–Darling tests were used to analyze the normal distribution characteristics of the numerical variables.

The independent sample *t*-test was used to compare two independent groups with normally distributed numerical variables, and the Mann–Whitney *U* test was used to compare two independent groups with non-normally distributed numerical variables.

The one-way analysis of variance (ANOVA) test was used to compare more than two independent groups with normally distributed numerical variables, and the Kruskal–Wallis test was used to compare more than two independent groups with non-normally distributed numerical variables. In the event of the analyses involving parametric tests, the differences between the groups, where data was heterogeneous according to distribution, were evaluated with The Games–Howell test. On the other hand, in the event of the analyses involving non-parametric tests, the Dwass–Steel–Critchlow–Fligner test was used to evaluate the differences between the groups.

Pearson’s chi-squared and Fisher’s exact tests were used to compare the differences between categorical variables in 2 × 2 tables. Additionally, the Fisher–Freeman–Halton test was used to compare the differences between categorical variables in R × C tables.

Spearman’s correlation analysis was used to analyze the correlations between age, postoperative BMI and EWL values, fecal elastase levels, and the GIQLI scores.

Jamovi project (Jamovi, version 2.2.5, 2022, retrieved from https://www.jamovi.org) and JASP (Jeffreys’ Amazing Statistics Program, version 0.16.1, 2022, retrieved from https://jasp-stats.org) software packages were used in the statistical analysis. Probability (p) values of ≤ 0.5 were deemed to indicate statistical significance.

## Results

There were 17, 29, 21, and 15 patients in the OAGB, SG, TB, and SASI groups, respectively. There was no significant difference between the groups in terms of age and gender (*p* = 0.053 and *p* = 0.338, respectively) (Table 1). The preoperative BMI values of the patients in group SASI were significantly higher than those in group OAGB (*p* = 0.008) and group TB (*p* = 0.007).

**Table 1:** Demographic and baseline clinical characteristics of the study groups

		Groups			
	OAGB	SG	TB	SASI	*p*
	(*n* = 17)	(*n* = 29)	(*n* = 21)	(*n* = 15)	
Age (year) ^†^	43.7 ± 8.1	38.6 ± 11.1	46.6 ± 9.1	46.1 ± 12.2	0.053*
Sex ^‡^					
Female	12 (70.6)	18 (62.1)	13 (61.9)	13 (86.7)	0.338***
Male	5 (29.4)	11 (37.9)	8 (38.1)	2 (13.3)	
BMI ^†^	43.4 ± 4.4	46.5 ± 5.0	43.7 ± 4.9	50.3 ± 9.5	0.021*

^‡^, *n* (%); †, mean ± standard deviation; §, median [min–max]. *SG*, sleeve gastrectomy; *OAGB*, one anastomosis gastric bypass; *SASI*, single anastomosis sleeve ileal bypass; *TB*, transit bipartition; *BMI*, body mass index. *One-way ANOVA test. **Kruskal–Wallis *H* test. ***Pearson chi-square, Fisher’s exact, or Fisher Freeman Halton tests

The postoperative second-year follow-up data is given in Table 2. There was no significant difference between the groups in terms of morphometric data, including BMI, % Δ BMI, and EWL values (*p* = 0.088, *p* = 0.093, and *p* = 0.058, respectively).

**Table 2:** Postoperative changes regarding weight changes, gastrointestinal symptoms, GIQLI score, and fecal elastase values in the study groups

		Groups			
	OAGB (*n* = 17)	SG (*n* = 29)	TB (*n* = 21)	SASI (*n* = 15)	*p*
BMI ^†^	29.4 ± 3.6	28.8 ± 4.6	27.2 ± 4.7	32.3 ± 6.5	0.088*
% Δ BMI ^§^	− 30.8 [− 46.4 to − 22.6]	− 36.9 [− 51.7 to − 22.3]	− 38.3 [− 49.6 to − 23.1]	− 36.0 [− 47.9 to − 16.5]	0.093**
EWL % ^†^	74.6 ± 15.2	82.9 ± 18.2	90.6 ± 23.5	73.6 ± 21.3	0.058*
Gastrointestinal symptoms					
Abdominal pain ^‡^	6 (35.3) a	3 (10.3) b	5 (23.8) a.b	7 (46.7) a	0.035***
Flatulence ^‡^	12 (70.6) a	6 (20.7) b	16 (76.2) a	12 (80.0) a	< 0.001***
Diarrhea ^‡^	5 (29.4) a	1 (3.4) b	6 (28.6) a	4 (26.7) a	0.029***
Constipation ^‡^	4 (23.5)	5 (17.2)	1 (4.8)	3 (20.0)	0.400***
Reflux ^‡^	7 (41.2)	5 (17.2)	3 (14.3)	3 (20.0)	0.222***
GIQLI score ^§^	93.0 [53.0–135.0]	109.0 [54.0–136.0]	111.0 [53.0–139.0]	102.0 [59.0–132.0]	0.152**
Fecal elastase ^§^	232.6 [106.6–258.4]	237.4 [181.5–279.8]	237.9 [138.4–273.0]	229.2 [131.2–257.1]	0.361**
Moderate EPI (100 to 200 µg/g) ‡	4 (23.5)	5 (17.2)	3 (14.3)	3 (20.0)	0.886***
Normal (> 200 µg/g) ‡	13 (76.5)	24 (82.8)	18 (85.7)	12 (80.0)	

^‡^, *n* (%); †, mean ± standard deviation; §, median [min–max]. *SG*, sleeve gastrectomy; *OAGB*, one anastomosis gastric bypass; *SASI*, single anastomosis sleeve ileal bypass; *TB*, transit bipartition; *BMI*, body mass index; *EWL*, excess weigh loss; *GIQLI*, gastrointestinal quality of life index; *EPI*, exocrine pancreatic insufficiency. *One-way ANOVA test. **Kruskal–Wallis *H* test. ***Pearson chi-square, Fisher’s exact, or Fisher Freeman Halton tests

On the other hand, there were significant differences between the groups in terms of the rates of postoperative gastrointestinal complaints (Table 2). Accordingly, the rate of patients with abdominal pain in group SG was significantly lower than in groups OAGB and SASI (*p* = 0.035). Similarly, the rates of patients with flatulence and diarrhea in group SG were significantly lower than those in the other three groups (*p* < 0.001 and *p* = 0.029, respectively). There was no significant difference between the groups in terms of other gastrointestinal complaints (*p* > 0.05).

There was no significant difference between the groups in terms of GIQLI scores and fecal elastase levels (*p* = 0.152 and *p* = 0.361, respectively) (Table 2).

The grouping made based on fecal elastase levels revealed no patient with severe EPI in any of the groups. The rates of patients with moderate EPI in groups OAGB, SG, TB, and SASI were 23.5%, 17.2%, 14.3%, and 20.0%, respectively. Accordingly, there was no significant difference between the groups in terms of the rate of patients with moderate EPI (*p* = 0.886).

There was also no significant difference in the GIQLI scores between the patients with moderate EPI and normal exocrine pancreatic function regardless of the group (*p* > 0.05) (Table 3).

**Table 3:** Association of GIQLI scores with moderate EPI and normal exocrine pancreatic function in each bariatric/metabolic surgery group

	GIQLI score	p*
SG		
Moderate EPI (*n* = 5)	79 [71–133]	0.470
Normal exocrine pancreatic function (*n* = 24)	109.5 [54–136]	
OAGB		
Moderate EPI (*n* = 4)	110.5 [53–135]	0.308
Normal exocrine pancreatic function (*n* = 13)	89 [65–126]	
TB		
Moderate EPI (*n* = 3)	56 [53–120]	0.070
Normal exocrine pancreatic function (*n* = 18)	116 [72–139]	
SASI		
Moderate EPI (*n* = 3)	115 [90–117]	0.386
Normal exocrine pancreatic function (*n* = 12)	102 [59–132]	

^§^, median [min–max]. *SG*, sleeve gastrectomy; *OAGB*, one anastomosis gastric bypass; *SASI*, single anastomosis sleeve ileal bypass; *TB*, transit bipartition; *GIQLI*, gastrointestinal quality of life index. *Mann–Whitney *U* test

The correlation analysis of the GIQLI scores with age, postoperative morphometric data, and fecal elastase values of the patients with different bariatric/metabolic surgeries showed no significant differences (*p* > 0.05) (Table 4).

**Table 4:** Correlation analysis of GIQLI with age, postoperative morphometric data, and fecal elastase values of the patients with different bariatric surgeries

	GIQLI score	
	*r*	*p*
OAGB		
Age	− 0.183	0.483
Postoperative BMI	− 0.253	0.327
% Δ BMI	− 0.361	0.155
EWL %	0.272	0.291
Fecal elastase	− 0.121	0.642
SG		
Age	0.047	0.809
Postoperative BMI	− 0.198	0.303
% Δ BMI	− 0.162	0.400
EWL %	0.149	0.440
Fecal elastase	0.327	0.084
TB		
Age	0.330	0.144
Postoperative BMI	0.330	0.144
% Δ BMI	− 0.045	0.845
EWL %	− 0.239	0.296
Fecal elastase	0.072	0.755
SASI		
Age	0.157	0.576
Postoperative BMI	− 0.204	0.466
% Δ BMI	− 0.261	0.348
EWL %	0.322	0.242
Fecal elastase	0.084	0.766

r, Spearman’s correlation coefficient. *SG*, sleeve gastrectomy; *OAGB*, one anastomosis gastric bypass; *SASI*, single anastomosis sleeve ileal bypass; *TB*, transit bipartition; *BMI*, body mass index; *EWL*, excess weight loss; *GIQLI*, gastrointestinal quality of life index

## Discussion

The findings of this study revealed that the rates of patients with moderate EPI diagnosed based on the fecal elastase levels measured during the second-year follow-up ranged from 14.3 to 23.5%, depending on the type of bariatric/metabolic surgery patients had. There was no severe EPI in the study cohort. Although postoperative complaints were significantly less in the SG group, the GIQLI scores were not significantly associated with the fecal elastase levels and bariatric/metabolic surgery types.

The relationship between the prevalence of EPI and the types of bariatric/metabolic surgery has been addressed in the literature. The EPI prevalence reported in these studies ranged between 4.3 and 75%, depending on the clinical characteristics of the patients and the types of surgical procedures performed [1, 3–5, 16]. It is generally accepted that patients with resectional types of surgical procedures or surgeries with altered anatomy are more prone to developing EPI [1, 3]. Decreased contact time between ingested food and pancreatic enzymes might be the major pathophysiological mechanism for experiencing EPI more frequently after different surgery types [5]. Uribarri-Gonzalez et al. [1] and Moore et al. [5] compared the rates of patients who developed EPI following different types of surgeries, namely SG, Roux-en-Y gastric bypass, and biliopancreatic diversion with duodenal switch and found that most patients with the biliopancreatic diversion with duodenal switch developed EPI in the postoperative period. Additionally, they found that patients with SG had the lowest rate of EPI, contrary to the results of this study [1, 5]. In comparison, the group with the lowest rate of EPI in this study was the TB group. On the other hand, Roux-en-Y gastric bypass and biliopancreatic diversion with duodenal switch were not performed in this study. Therefore, the relatively low rates of EPI found in this study may be attributed to the sample size and the types of surgical procedures performed.

The quality of life after bariatric/metabolic surgery is another controversial matter. Several tools, such as GIQLI,36-item Short Form Health Survey (SF-36), BAROS, and the Moorehead-Ardelt Quality of Life Questionnaire, are used to evaluate patients’ postoperative quality of life [6, 13, 22]. Two systematic reviews and meta-analyses found no significant difference between patients with SG and Roux-en-Y gastric bypass in the quality-of-life scores, regardless of the tool used [13, 22]. Nickel et al. [11] showed that the differences between the patients who had SG and Roux-en-Y gastric bypass in the GIQLI scores lost their significance as the follow-up time increased. Singla et al. [12] reported the mean GIQLI score of their cohort as125 ± 13.1 out of 144 at a median follow-up duration of 36 months following OAGB, which they interpreted as a reflection of the good health status of their cohort. In comparison, the median GIQLI scores ranged from 93 to 111 in the study groups included in this study, which are compatible with the findings of various other studies [7, 11, 15]. The subjective assessment of these scores in a heterogeneous study cohort may lead to biases. Although there was no significant difference between the groups in GIQLI scores, the patients in group OAGB had relatively lower scores than the other three groups. Guman et al. [6] showed an improvement in the postoperative GIQLI scores compared to the preoperative GIQLI scores. In line with the findings of Singla et al. [12], no significant correlation was found in this study between age, current BMI values, percent BMI and EWL changes, fecal elastase levels, and GIQLI scores. The reasons for the controversy between the respective findings in the literature might be multi-factorial, including the length of the interval between the surgery and the assessment, the amount of weight loss, and the type of surgery. Therefore, prospective studies with larger samples are needed.

The length of the interval between the surgery and the detection of EPI and the assessment by GIQLI varies between different studies. These studies generally utilized longer mean follow-up durations, such as 24 to 36 months, for the diagnosis of EPI, contrary to few other studies with shorter follow-up durations, such as 6 months [3, 6, 16]. In Moore’s study, the median time from surgery to EPI diagnosis was 9.46 years [5]. In comparison, the EPI and quality of life were assessed in this study in the postoperative second year, in line with the idea that there should be enough time for the stabilization of the patients in terms of weight and gastrointestinal symptoms in order to assess EPI or quality of life [9, 23].

### Limitations of the Study

Apart from the prospective and comparative design of the study, which can be considered its strengths, there were also some limitations to this study. First, the sample size was relatively small. Secondly, preoperative and postoperative fecal elastase levels and GIQLI scores were not compared. The heterogeneity of patients’ clinical characteristics and types of bariatric/metabolic surgery they had can be considered other limitations that prevented making healthier generalizations based on the results of the study. Additionally, the maladaptive eating habits associated with the symptomatology of the patients were not studied. Furthermore, the relationship between the clinical symptoms and EPI could not be analyzed, given the small number of patients presenting each symptom.

## Conclusion

In conclusion, EPI rates varied depending on the type of surgery. However, no significant relationship was found between the rates of patients with EPI, patients’ GIQLI scores, and the types of surgical procedures patients had. Large-scale studies are needed to demonstrate the possible impact of EPI on the quality of life in patients with bariatric/metabolic surgery.
